# The Crystallization and Melting Behavior of Neodymium-Based Butadiene Rubber Blends

**DOI:** 10.3390/polym16030342

**Published:** 2024-01-26

**Authors:** Xiaofan Li, Xiaohu Zhang, Huan Ji, Yanxing Wei, Xinzheng Xie, Wenbin Zhu, Jifu Bi

**Affiliations:** 1 Huangpu Institute of Materials, Centers for Aircraft Science, Guangzhou 510700, China; lixiaofan@ciac.ac.cn (X.L.); jihuan@ciac.ac.cn (H.J.); weiyanxing@ciac.ac.cn (Y.W.); xiexinzheng@ciac.ac.cn (X.X.); zhuwenbin@ciac.ac.cn (W.Z.); 2 Changchun Institute of Applied Chemistry Chinese Academy of Sciences, Changchun 130102, China

**Keywords:** neodymium-based butadiene rubber, dilution effect, equilibrium melting point, crystalline structure

## Abstract

This study investigates the influence of poly(butadiene-isoprene) copolymer rubber (BIR) and TDAE oil on the crystallization and melting behavior of neodymium-based butadiene rubber (Nd-BR). The study demonstrates that the melting points of Nd-BR and its blends decrease with lower crystallization temperatures. Below the critical crystallization temperature (*T*_c,c_), the melting behavior shows dual peaks in distinct temperature ranges, which are attributed to different spherulitic sizes. The addition of BIR or TDAE oil lowers the *T*_c,c_, with TDAE oil exerting a more substantial effect. These diluents mainly influence the nucleation temperature and crystallinity level of Nd-BR while having a minimal effect on the crystallization mechanism. A master curve, which overlaps for various samples, is developed by correlating the peak melting temperature (*T*_m,peak_) with the *T*_c_. This curve facilitates a quantitative assessment of the effects of BIR and TDAE oil on Nd-BR, highlighting the greater influence of TDAE oil on the crystalline structure compared with BIR at equivalent mass fractions. By applying the Lorentz equation and multi-peak fitting, a relationship between the melting points and crystallization temperatures is established, enabling the calculation of the equilibrium melting points (*T*_m_^0^) for different samples. The findings show a reduction in the *T*_m_^0^ due to the diluents; specifically, the *T*_m_^0^ is approximately 0 °C for pure Nd-BR, and it decreases to −4.579 °C and −6.579 °C for samples with 50 PHR TDAE oil and 60 wt.% BIR, respectively.

## 1. Introduction

Neodymium-based butadiene rubber (Nd-BR) is prepared via polymerization reaction with the compound of the rare earth element Nd as the catalyst and butadiene as the raw material. The cis-1,4-structure content of Nd-BR is greater than 95 wt.%, and it has the characteristics of high linearity, less branching and no gel [1,2]. At present, Nd-BR is developing in the direction of narrow molecular weight distribution and high Mooney viscosity [3]. However, the thermal motion of rubber molecules is weakened under low temperatures, and Nd-BR is more susceptible to freezing and crystallization due to the high cis-structure content and regular molecular chain structure, thus gradually losing elasticity, which is not conducive to the application of rubber in the field of temperature [4]. Consequently, knowledge of the crystallization kinetics is crucial for the application and development of elastomers and their compounds.

The crystallization and melting behavior of High-cis-1,4-Polybutadienes rubber has been studied using different methods [5,6]. For instance, FEIO [7] has studied the isothermal crystallization kinetics of cis-1,4-polybutadiene by using 1 H pulsed high-resolution FT-NMR. It was found that for undercoolings up to 30 K, the primary nucleation of the PB is predominantly heterogeneous, while for higher degrees of undercooling from Δ30 K–50 K, the nucleation is homogeneous. And there were two limiting temperatures that could be estimated via further analysis of the temperature dependence of the crystallization rate: the equilibrium melting temperature T_m_^0^ = 278 K, and the temperature where all local motions cease, T^0^ = 133 K. At these temperatures, crystallization is infinitely slow. Yang [8] investigated the crystallization morphology of Nd-BR and revealed a close connection between the nucleation mechanism and temperature. Above −20 °C, Nd-BR cannot nucleate homogeneously and only grows crystals through heterogeneous nucleation. In the temperature range of −22 to −33 °C, both homogeneous and heterogeneous nucleation mechanisms coexist, while at −35 to −65 °C, homogeneous nucleation takes precedence. These distinct nucleation mechanisms result in various crystalline structures. Nd-BR can manifest six distinct crystalline morphologies between −10 and −80 °C: larger spherulites at higher temperatures, two types of spherulites around −30 ± 10 °C, smaller spherulites at −40 to −60 °C with crystals growing in bundles from the center, a combination of small spherulites and bundled structures at −60 to −80 °C, and large finely structured spherulites at even lower temperatures. Maria Laura Di Lorenzo studied the melting behavior of cis-1,4-polybutadiene in detail [9]. After isothermal crystallization at −26 °C followed by cooling, up to three peaks can be observed in the melting curve of the sample, which indicates the samples provide a three-phase structure composed of a mobile amorphous, crystallinity and rigid amorphous. It is also highlighted in another work by Maria Laura Di Lorenzo that after polybutadiene rubber crystallization, a three-phase structure exists when cooled to the glass transition temperature, leading to distinct melting behaviors [10]. The crosslinking reaction restricts molecular mobility, thereby causing a noteworthy reduction in both the crystallization temperature and crystallinity [11,12]. Moreover, the crystallization phenomena in polymer blends were studied for various compositions. In the polymer blends, crystallinity may be affected by the other polymer [13]. In the PS/PP blends, the crystallinity of the prepared blends significantly decreases compared to neat PP, while the microfibrillar morphology induces homogeneous crystallization with small crystallites, leading to a decrease in the crystallization temperature, crystallinity, and a lower melting temperature [14]. Fenni studied the nucleation and crystallization of finely dispersed semicrystalline polymeric droplets in immiscible matrices and reported the role of interfacial roughness in nucleation [15]. In the miscible polymer blends, crystallization may be different due to the phase segregation. When both components are crystallizable, concentrically growing or interpenetrating spherulites have recently been reported to occur via simultaneous crystallization [16,17,18,19]. When a component is amorphous, the inclusion or exclusion of the amorphous component is dominated by the relationship between the crystallization rate and the molecular diffusion rate according to the Keith–Padden theory [20]. When the crystallization rate is sufficiently low and the mobility is sufficiently high, the amorphous component can be excluded from the interlamellar region, even in miscible blends. Meanwhile, the amorphous component in miscible polymer blends usually decreases the crystallization rates through the effect of the composition dependence of the glass transition temperature and through diluent effects.

In our previous research, the influence of both treated distillate aromatic extract (TDAE) oil and poly(butadiene-isoprene) copolymer rubber (BIR) on the crystallization kinetics of Nd-BR was studied [21]. Due to different crystallization temperatures for different Nd-BR/BIR or Bd-BR/TDAE oil compounds, the occurrence of dual-peak melting during crystallization at −35 °C to 66 °C affects the calculation of the equilibrium melting points (*T*_m_^0^), indicating alterations in the crystalline structure and melting behavior in different temperature ranges. To gain deeper insights into the influence of the temperature and diluents on Nd-BR crystallization and melting behavior, this study investigated the effects of TDAE oil and BIR on Nd-BR crystallization and melting, and the *T*_m_^0^ was calculated for different samples. The objective was to elucidate the factors influencing Nd-BR crystallization and to provide a theoretical foundation for the development and application of Nd-BR rubber.

## 2. Materials and Methods

### 2.1. Raw Materials

Nd-BR with a grade of BR 9101N (density: 0.92 g/cm^3^; *M*_n_: 93,380 g/mol, molecular weight distribution (MWD): 3.719) was obtained from Dushanzi Petrochemical Co., Ltd. (Karamay, China). TDAE oil (Density: 0.953 g/cm^3^; carbon type distribution for aromatic/naphthenic/paraffinic is 23%/39%/38%) was supplied by Hansheng Chemical Co., Ltd. (Ningbo, China). BIR (isoprene content: 18 wt.%; cis-structure%: 97%; *M*_n_: 126,468 g/mol and MWD: 2.72) was synthesized in-house.

### 2.2. Sample Preparation

The method for preparing the Nd-BR/TDAE oil blends: A total of 200 g of Nd-BR rubber was plasticized in an open mill at a temperature of 50 °C. After the rubber was sufficiently masticated, TDAE oil was added incrementally, each time at a dosage of 10 PHR. Following thorough mixing, samples were retained. Oil-extended rubbers with oil contents of 10 parts per hundreds of rubbers (PHR), 20 PHR, 30 PHR, 40 PHR, and 50 PHR were obtained and designated BR-10T, BR-20T, BR-30T, BR-40T, and BR-50T, respectively.

The method for preparing the BR/BIR blends: BR and BIR were dissolved in hexane solvent according to their respective proportions. The homogeneous rubber solution was then poured into ethanol for coagulation. After air-drying for 12 h in a fume hood, the samples were dried in a vacuum oven at 60 °C and 1000 Pa for 24 h. The blended rubbers containing 100 wt.%, 80 wt.%, 60 wt.%, 40 wt.%, 20 wt.%, and 0 wt.% BR were labelled BR100, BR80, BR60, BR40, BR20 and BIR, respectively.

### 2.3. Testing and Characterization

The crystallization behavior testing was conducted using a Differential Scanning Calorimeter (DSC25, TA, New Castle, DE, USA) and analyzed using the TRIOS software v5.2.2.47561. The average mass of the samples was 10 mg, and they were cooled from 30 °C to −80 °C at rates of 5 or 10 °C/min and then isothermally maintained at the designated temperature for 1 min before being heated to 30 °C at a rate of 10 °C/min. The entire test procedure was carried out in a nitrogen atmosphere.

For the crystallization kinetics testing, the samples were rapidly cooled to the specified crystallization temperature and held isothermally for 30 min. Subsequently, they were subjected to a controlled heating process of up to 30 °C at a rate of 10 °C/min. This cyclic process was repeated at various crystallization temperatures, with intervals ranging from 2 to 4 °C between each temperature increment.

## 3. Results

### 3.1. Crystallization Behavior of Nd-BR

**Figure 1:**
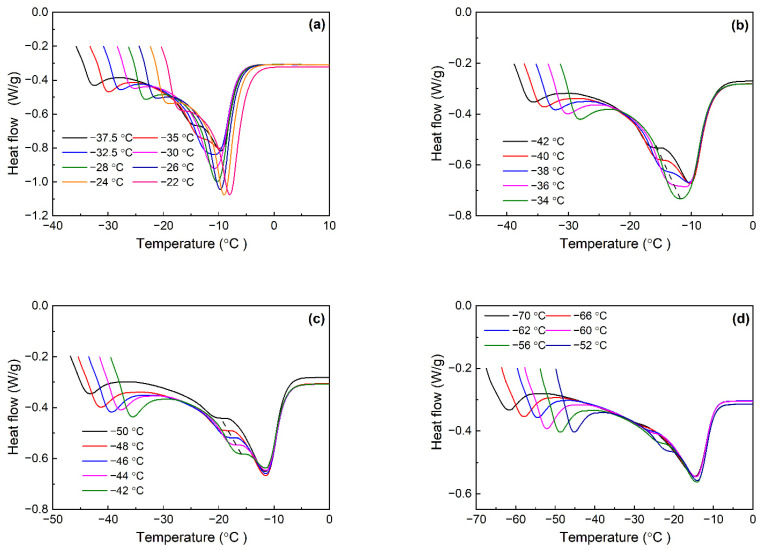
Melting curves of Nd-BR/BIR compounds after isothermal crystallization at different temperature: (**a**) BR100; (**b**) BR80; (**c**) BR60; and (**d**) BR40.

Table 1 lists the crystallization and melting parameters of various Nd-BR composites during the constant-rate cooling and heating processes. Isothermal crystallization was conducted at various temperatures, depending on the crystallization temperatures of the different samples. Following the completion of crystallization, the samples were heated at a rate of 10 °C/min to generate melting curves for the crystals formed at different temperatures. Figure 1 illustrates the melting curves of Nd-BR and its blends with BIR at various temperatures.

It is evident that Nd-BR exhibits a solitary melting peak when crystallized at temperatures ≥ −32.5 °C. However, below this temperature, the melting curves revealed a distinct double-peak pattern, signifying the presence of diverse crystalline structures. This double-peak configuration leads to the broadening of the melting peaks of Nd-BR, with the lower-temperature melting peak shifting downward as the crystallization temperature decreases. Conversely, the temperature of the high-temperature melting peak remains relatively stable as the crystallization temperature decreases.

Upon comparing the melting curves of the Nd-BR/BIR composites with pure butadiene rubber (Figure 1b–d), it is apparent that an increase in the BIR content results in a reduction in the peak heat flow of the melting peaks, which is indicative of a decreased crystallization extent. Furthermore, except for the BR80 sample crystallized at 34 °C, all the samples exhibited dual melting peaks after crystallization at the corresponding temperatures. However, the associated crystallization temperatures fall within the range of −36 to −38 °C, which is lower than that of the pure butadiene rubber BR100. Analogous to Nd-BR100, the lower-temperature melting peak temperature decreases with a decreasing crystallization temperature as indicated by the dashed line, while the higher-temperature melting peak temperature remains nearly unchanged. For BR40 crystallized below −60 °C, the lower-temperature melting peak becomes feeble or even disappears upon melting.

**Figure 2:**
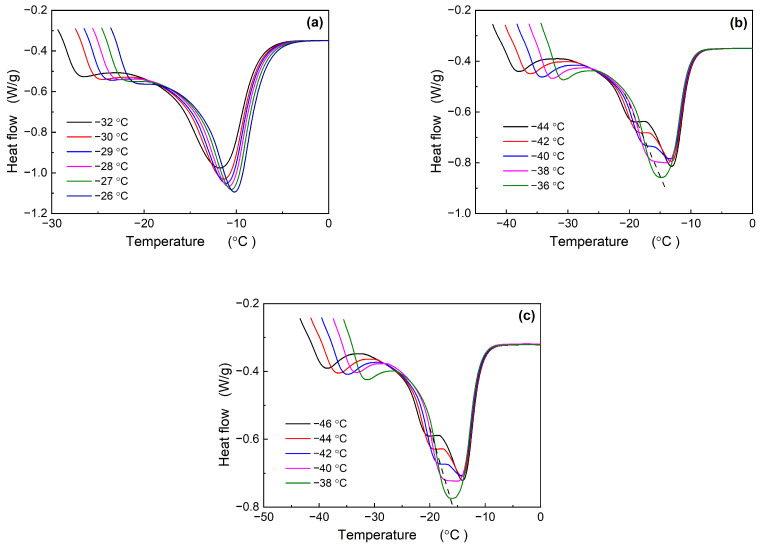
Melting curves of Nd-BR/TDAE oil compounds after isothermal crystallization at different temperatures: (**a**) BR-10T; (**b**) BR-30T; and (**c**) BR-50T.

Figure 2 illustrates the effect of TDAE oil on the melting behavior of Nd-BR following isothermal crystallization. The BR-10T sample, characterized by a lower TDAE oil content and a higher crystallization temperature, consistently displays single-peak melting curves across all the measurements. However, as the crystallization temperature decreases, a discernible trend of increasing peak width is observed. Elevated TDAE oil content results in a reduction in the crystallization temperature and a transition from a single melting peak to dual peaks: BR30T begins to manifest dual melting peaks at temperatures ranging from −38 to −40 °C, while BR-50T exhibits dual peaks at −40 to −42 °C. In contrast, the pure Nd-BR samples exhibit dual peaks between −32.5 and −35 °C. Consequently, the introduction of TDAE oil shifts the crystallization temperature for the emergence of dual melting peaks to lower temperature ranges.

**Figure 3:**
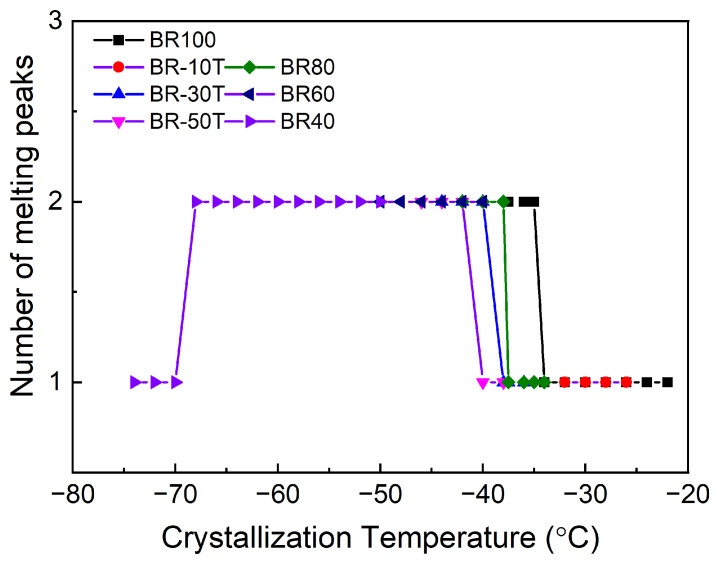
The number of melting peaks of different samples according to the crystallization temperature.

Figure 3 depicts the correlation between the number of melting peaks and the crystallization temperature for different samples after crystallization at varying temperatures. Notably, the transition in the number of melting peaks predominantly occurs within the temperature range of −35 to −40 °C across the different samples. When the crystallization temperature reaches −70 °C, the melting peak reverts to a singular configuration.

As documented in the literature [8], the crystallization behavior of BR exhibits specific characteristics in different temperature ranges. Above −20 °C, BR undergoes crystallization primarily through heterogeneous nucleation. In the temperature range of approximately −22 to −33 °C, both homogeneous and heterogeneous nucleation mechanisms coexist, leading to a mixture of crystalline structures. From −35 to −65 °C, homogeneous nucleation becomes the predominant mechanism. These distinct nucleation mechanisms result in diverse crystalline structures. As the crystallization temperature decreases, the spherulitic morphology undergoes a transition from larger to smaller spherulites, with coexisting large/small spherulites or small spherulites/bundled structures occurring at intermediate temperatures.

However, it is noteworthy that the critical temperatures associated with different crystal types, as reported in the literature, do not align entirely with the temperatures of the melting peaks observed in the DSC curves in our experiments. For instance, the literature suggests the coexistence of large/small spherulites within the −22 to −35 °C range, whereas in our experimental data, dual melting peaks for pure butadiene rubber are only observed at −35 °C, and at even lower temperatures for the Nd-BR blends. This disparity could be attributed to variations in the testing methods, the sample types employed, and the potential influence of sample mixtures on the resulting crystalline structure.

### 3.2. Analysis of Melting Behavior of Nd-BR and Its Composites

**Figure 4:**
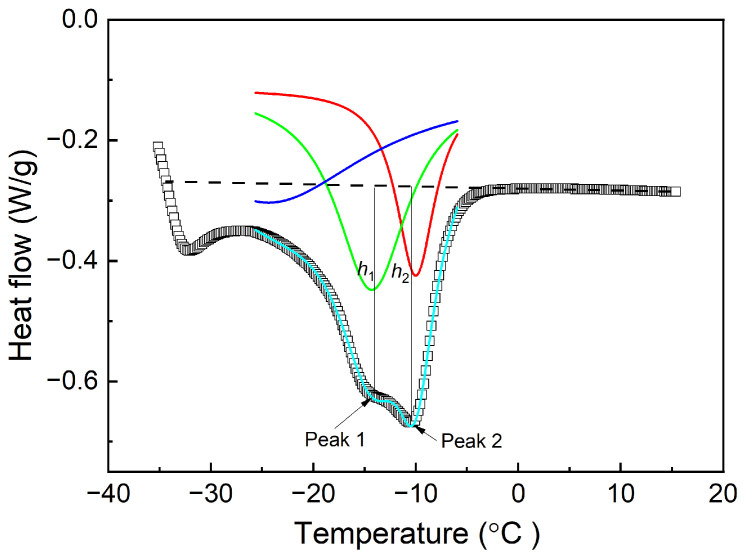
Analysis methods concerning the melting curves of the Nd-BR compounds.

To facilitate a more precise analysis of the influence of the crystallization temperature on the occurrence of dual melting peaks, a multi-peak fitting approach was employed to model the melting curves. This method allows for the accurate determination of the melting points at varying crystallization temperatures. The specific methodology used for this analysis is illustrated in Figure 4, the original melting curve containing two peaks was divided into two separate curve peaks as shown in the red and green lines. The curve obtained by fitting the red line and green line is shown in the fluorescence blue curve, which basically overlaps with the original curve, indicating that multi-peak fitting approach is credible. The melting points corresponding to the low- and high-temperature melting peaks are designated as *T*_m,1_ and *T*_m,2_, respectively. Furthermore, the peak heights, which serve as indicators of the relative content of the crystalline structures associated with each melting peak, are represented as *h*_1_ and *h*_2_ for the low- and high-temperature peaks, respectively. 

#### 3.2.1. Calculation and Analysis of the Equilibrium Melting Point of Nd-BR

The melting point (*T*_m_) of a polymer during crystallization and melting is related to the lamellae thickness as follows: $T_{m}={T_{m}}^{0}(1-\frac{2\sigma_{e}}{l∆h})$, where *T*_m_ and the equilibrium melting point (*T*_m_^0^) represent the melting points when the lamellae thickness is finite (*l*) and infinite (∞), respectively. ∆h is the heat of fusion per unit volume and $\sigma_{e}$ is the surface energy. As detailed in Table 1, the melting points decrease with increasing TDAE oil and BIR contents. This observation implies that both components act as diluents for the Nd-BR molecular chains, leading to an expansion of the intermolecular spacing and impeding the crystal growth, thereby reducing the lamellae thickness. *T*_m_^0^ can be determined by identifying the intersection of the *T*_m_~*T*_c_ curve with the line *T*_m_ = *T*_c_.

**Figure 5:**
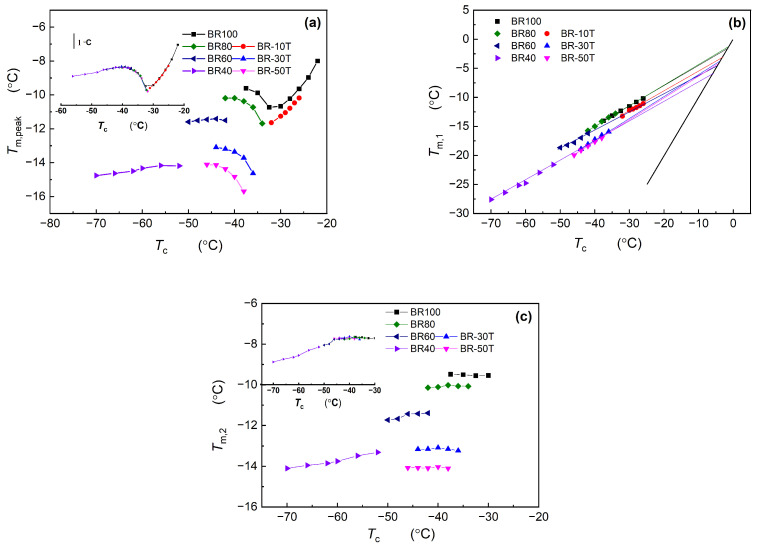
Dependence of the melting point (*T*_m,peak_, *T*_m,1_ and *T*_m,2_) on the crystallization temperature.

Figure 1 and Figure 2 clearly illustrate that Nd-BR and its composites exhibit dual melting peaks across a broad temperature spectrum. Plotting the peak temperature (*T*_m,peak_) against the crystallization temperature (*T*_c_), as depicted in Figure 5a, reveals that *T*_m,peak_~*T*_c_ does not adhere to a linear relationship, which can be observed intuitively in the subfigure. This complexity poses challenges in determining the equilibrium melting points of the samples using this approach. This variation arises from the formation of distinct crystalline structures at different temperatures, leading to fluctuations in the maximum melting temperature. Nevertheless, the *T*_m,peak_~*T*_c_ curves for the different samples exhibit analogous trends within the same crystallization temperature range. For instance, at higher temperatures, *T*_m,peak_ experiences a rapid increase with increasing *T*_c_; within the temperature range of −35 to −33 °C, *T*_m,peak_ decreases as *T*_c_ increases; and at lower temperatures, *T*_m,peak_ registers a gradual rise with elevated *T*_c_. By leveraging the *T*_m,peak_~*T*_c_ curve of pure Nd-BR as a reference, a master curve can be derived by aligning the curves of various samples, as depicted in the inset of Figure 5a. This correlation between the *T*_m,peak_ and crystalline structure suggests that TDAE oil or BIR does not induce alterations in the crystalline structure of Nd-BR but shifts the temperature range at which different crystalline structures form.

Employing the analytical method illustrated in Figure 4, the values of *T*_m,1_ and *T*_m,2_ were determined and plotted against *T*_c_, as illustrated in Figure 5b,c. Evidently, the *T*_m,1_~*T*_c_ plot exhibits a well-defined linear relationship, indicating that the crystalline structure corresponding to *T*_m,1_ predominates under various isothermal conditions. The equilibrium melting points (*T*_m_^0^) of different samples can be determined by identifying the intersection of the *T*_m,1_–*T*_c_ plot with the line *T*_m_ = *T*_c_ (black line), as presented in Table 1. For pure butadiene rubber BR100 and BR80, *T*_m_^0^ is approximately 0 °C, which is consistent with the findings reported in the literature [8]. In contrast, the equilibrium melting points of BR60 and BR40 are −4.922 °C and −6.579 °C, respectively. Notably, TDAE oil exerts a more pronounced solvating effect on Nd-BR than macromolecular BIR. Only 10 PHR (9.1 wt.%) of TDAE oil is sufficient to lower *T*_m_^0^ to −3.194 °C, and further increases in the TDAE oil content lead to a gradual reduction in the equilibrium melting point.

Figure 5c highlights the relationship between *T*_m,2_ and the crystallization temperature, which can be observed more intuitively in the subfigure. *T*_m,2_ remains relatively constant for different samples when isothermally crystallized above −50 °C. Additionally, as the content of TDAE oil or BIR increases, *T*_m,2_ decreases across various samples, signifying that the crystalline structure corresponding to *T*_m,2_ is primarily influenced by the composition of Nd-BR and is less dependent on the crystallization temperature. Below −50 °C, *T*_m,2_ experiences a gradual increase with an increasing crystallization temperature, mirroring the behaviors of *T*_m,peak_ and *T*_m,1_. This phenomenon can be attributed to diffusion-controlled crystallization at lower temperatures, where temperature plays a pivotal role in dictating crystal growth and significantly affects different structural formations. By shifting the *T*_m,2_, a curve is generated, as shown in the inset of Figure 5c, featuring an inflection point around −50 °C. This inflection suggests that temperature is the primary determinant of the crystal structure associated with *T*_m,2_, whereas the solvating influence of TDAE oil or BIR on Nd-BR predominantly affects the lamellae thickness and perfection.

#### 3.2.2. Height of Melting Peak

Both TDAE oil and BIR exert a diluting and inhibitory influence on the crystallization of Nd-BR, resulting in a reduction in the crystallization intensity. However, assessing the degree of crystallization within the same temperature range via integration poses challenges, owing to the varying crystallization temperatures of different materials. Therefore, the heights of the different melting peaks in the multi-peak melting curves (as depicted in Figure 4) are employed to represent the content of the corresponding crystalline structures.

**Figure 6:**
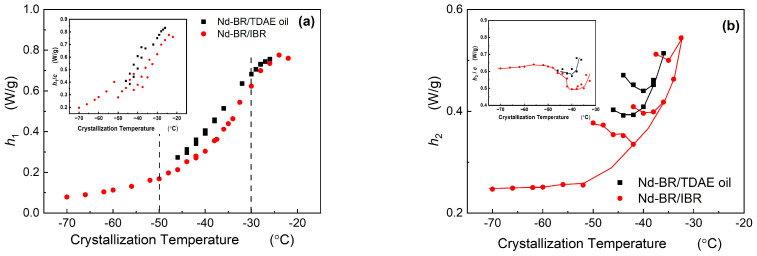
Dependence of the peak height on the crystallization temperature.

Figure 6 illustrates the relationship between the peak height and crystallization temperature. As shown in Figure 6a, *h*_1_ decreases as the crystallization temperature decreases. The *h*_1_~*T*_c_ behavior of the Nd-BR/BIR composites follows a similar trend, and likewise, the *h*_1_~*T*_c_ curves of the Nd-BR/TDAE oil samples exhibit a consistent pattern, irrespective of the oil content. Nevertheless, this may appear counterintuitive because various samples possess different concentrations of Nd-BR, which should logically affect the extent of the crystallization. By normalizing the *h*_1_/c, where c is the percentage content of the sample (as shown in the inset of Figure 6a), the overarching trend of the *h*_1_ decreases with a decreasing crystallization temperature. The consistent peak height *h*_1_, coupled with the inclusion of diluent materials, results in a reduction in the corresponding crystallization temperature, signifying that the diluents impede crystallization. TDAE oil produced a similar effect, albeit with a more pronounced reduction in the crystallization temperature.

Figure 6b shows the variation in the *h*_2_ with respect to the crystallization temperature. It is evident that within the −30 to −50 °C range, the peak height increases as the temperature decreases during low-temperature crystallization, aligning with the trend observed in the melting point changes. This suggests that lower temperatures promote the growth of the corresponding crystal type, thereby increasing the extent of the crystallization. In addition, *h*_2_/c has a better correlation with *T*_c_ as shown in the sub-figure, indicating that h_2_ has a close relationship with the absolute content of Nd-BR(*h*_2_/c). Xu Yang et al. [8] observed through microscopy that both larger and smaller spherulites coexist within the −25 to −37.5 °C range, while temperatures ranging from −37.5 to −55 °C are characterized by the prevalence of smaller spherulites. These divergent spherulitic morphologies are intimately connected to the crystallization nucleation mechanism. By integrating the relationships among the spherulite structure, nucleation mechanism, and temperature, it can be inferred that the *h*_1_ in this experimental context corresponds to larger spherulites, primarily resulting from heterogeneous nucleation. In contrast, the *h*_2_ corresponds to smaller spherulites that predominantly originate from homogeneous nucleation. Consequently, the DSC data suggest that the Nd-BR composites exhibit the coexistence of larger and smaller spherulites over a broad crystallization temperature range. As the crystallization temperature decreases, the nucleation growth transitions from heterogeneous to homogeneous nucleation, leading to a decrease in the content of larger spherulites and an increase in smaller spherulites.

### 3.3. Analysis of the Impact of Diluents on Nd-BR Crystallization

The influence of TDAE oil and BIR on the crystallization of Nd-BR reveals that the dilution effect of the secondary component concurrently results in a decrease in the crystallization temperature, melting point, and equilibrium melting point. However, the specific effects of different diluents and their concentrations on crystallization vary. As illustrated in Figure 5, different diluents induce alterations in the crystallization temperature and melting point; however, the underlying crystallization mechanism and structure remain relatively unaltered. The *T*_m,peak_~*T*_c_ and *T*_m,2_~*T*_c_ curves for all the diluents can be harmonized by shifting them to create a master curve. Hence, the extent of the crystallization influence exerted by distinct materials can be characterized by the shift in the crystallization temperature (horizontal shift, *T*_c_) and melting point (vertical shift, *T*_m_) resulting from the various diluents.

**Figure 7:**
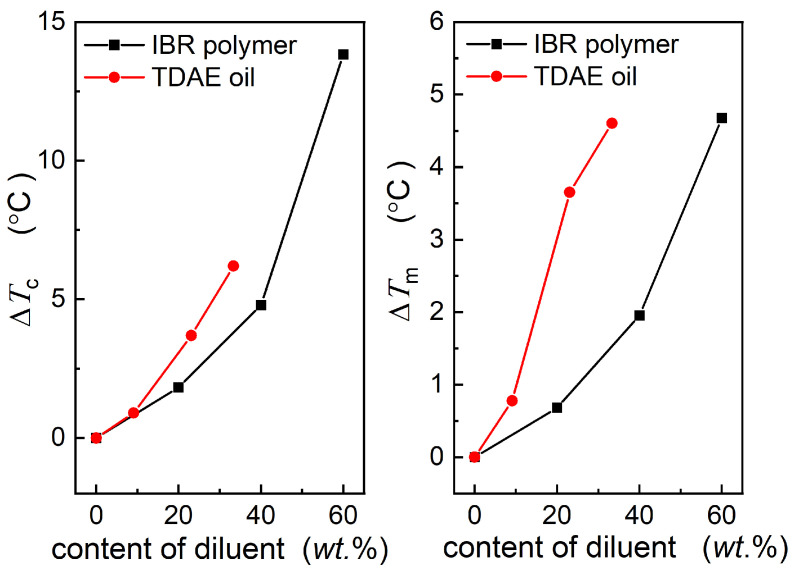
Dependence of the Δ*T*_c_ and Δ*T*_m_ on the diluent content, with pure Nd-BR as a reference.

Figure 7 depicts the relationship between the *T*_c_ and *T*_m_ with respect to the type and concentration of the diluents. It is evident that both the *T*_c_ and *T*_m_ increase as the diluent content increases, indicating a more pronounced effect on crystallization at higher concentrations. At equivalent mass fractions, TDAE oil exhibits considerably higher *T*_c_ and *T*_m_ values than BIR oil. This discrepancy can be attributed to TDAE oil being a small-molecule organic substance with enhanced compatibility with Nd-BR. The dispersion of oil molecules among the Nd-BR molecular chains results in increased intermolecular spacing, which consequently lowers the temperature of crystal nucleation. An elevated oil content leads to greater intermolecular distances, which diminishes the nucleation propensity. Furthermore, the potential induction of crystalline defects by TDAE may be more substantial, contributing to reduced lamellae thickness and a substantial decrease in the melting point.

In contrast, the macromolecular nature of BIR, coupled with its entropic elasticity, precludes it from dissolving thoroughly between the Nd-BR molecules as small molecules. Consequently, its influence on crystallization is less pronounced than that of the TDAE oil.

## 4. Conclusions

Investigation of the crystallization and melting behaviors of Nd-BR and its composites at various temperatures has revealed that diluents such as TDAE oil and BIR impede the nucleation and growth of larger spherulites, resulting in a reduction in the crystallization temperature, melting point, and equilibrium melting point. The crystallization temperature plays a pivotal role in determining the crystallization rate and structure. Above −35 °C, the isothermal crystallization of Nd-BR predominantly yields a solitary, larger spherulitic structure, leading to a single melting peak during the subsequent melting process. Within a broad temperature range spanning from −35 °C to 70 °C, both large and small spherulites may coexist, thereby giving rise to dual melting peaks. The melting points of the larger spherulites decrease as the crystallization temperature decreases, whereas those of the smaller spherulites exhibit less variability in response to temperature fluctuations. Nevertheless, it is noteworthy that the diluents primarily modulate the nucleation crystallization temperature and exert a modest influence on the crystalline structure. The principal curve, which represents the effect of temperature on crystallization, can be derived by shifting. Analysis of the *T*_m_~*T*_c_ principal curve suggests that the solvating effect and the effect of TDAE oil on the crystallization of Nd-BR surpass those of the BIR polymer. From the results above, the influence of TDAE and BIR on the crystallization temperature, crystalline structure and melting behaviors were analyzed, which is of significance when it comes to elaborating the mechanism of Nd-BR crystallization and instructing the application of Nd-BR at low temperatures.

## Figures and Tables

**Table 1:** Crystallization and melting behaviors of Nd-BR and its compounds.

	*T*_c,peak_ (°C)	Temperature Range of Crystallization (°C)	*T*_m_^0^ (°C)	*E*_a,t(1/2)_ (kJ/mol)
BR100	−37.44	−37.5~−24	−0.135	−264.3
BR80	−45.22	−42~−34	−0.0735	−232.8
BR60	−52.72	−50~−42	−4.922	−125.11
BR40	−68.67	−70~−52	−6.579	42.9
BR-10T	−41.04	−32~−26	−3.194	−191.4
BR-30T	−49.98	−44~−36	−3.572	−93.9
BR-50T	−55.23	−46~−38	−4.579	−50.6

## Data Availability

The data that support the findings of this work are available from the corresponding author on reasonable request.
